# Identification of Six New World *Leishmania* species through the implementation of a High-Resolution Melting (HRM) genotyping assay

**DOI:** 10.1186/s13071-014-0501-y

**Published:** 2014-11-14

**Authors:** Carolina Hernández, Catalina Alvarez, Camila González, Martha Stella Ayala, Cielo Maritza León, Juan David Ramírez

**Affiliations:** Grupo de Parasitología, Instituto Nacional de Salud, Bogotá, Colombia; Grupo de Investigaciones Microbiológicas - UR (GIMUR), Facultad de Ciencias Naturales y Matemáticas, Universidad del Rosario, Bogotá, Colombia; Centro de Investigaciones en Microbiología y Parasitología Tropical (CIMPAT), Departamento de Ciencias Biológicas, Facultad de Ciencias, Universidad de los Andes, Bogotá, Colombia

**Keywords:** *Leishmania*, Genotyping, Real-Time PCR, High-resolution Melting

## Abstract

**Background:**

Leishmaniases are tropical zoonotic diseases, caused by parasites from the genus *Leishmania*. New World (NW) species are related to sylvatic cycles although urbanization processes have been reported in some South American Countries such as Colombia. This eco-epidemiological complexity imposes a challenge to the detection of circulating parasite species, not only related to human cases but also infecting vectors and reservoirs. Currently, no harmonized methods have been deployed to discriminate the NW *Leishmania* species.

**Findings:**

Herein, we conducted a systematic and mechanistic High-Resolution Melting (HRM) assay targeted to HSP70 and ITS1. Specific primers were designed that coupled with a HRM analyses permitted to discriminate six NW *Leishmania* species. In order to validate the herein described algorithm, we included 35 natural isolates obtained from human cases, insect vectors and mammals. Our genotyping assay allowed the correct assignment of the six NW *Leishmania* species (*L. mexicana, L. infantum (chagasi), L. amazonensis, L. panamensis, L. guyanensis* and *L. braziliensis*) based on reference strains. When the algorithm was applied to a set of well-characterized strains by means of PCR-RFLP, MLEE and monoclonal antibodies (MA) we observed a tailored concordance between the HRM and PCR-RFLP/MLEE/MA (KI = 1.0). Additionally, we tested the limit of detection for the HRM method showing that this is able to detect at least 10 equivalent-parasites per mL.

**Conclusions:**

This is a rapid and reliable method to conduct molecular epidemiology and host-parasite association studies in endemic areas.

## Findings

Leishmaniasis is a tropical infection caused by the kinetoplastid parasites of the genus *Leishmania*, and is naturally transmitted to humans and among reservoirs by sandfly bites [1]. With a worldwide distribution and a large number of clinical cases, Leishmaniasis affects around 12 million individuals and 350 million are at risk of contracting the disease representing a serious issue in public health [2]. Additionally, control strategies against the disease are a challenge due to its eco-epidemiological complexity, involving sylvatic and domestic multi host-pathogen interactions (humans as well as a wide range of mammalian hosts). Recently, armed conflicts, increased tourism, natural disasters, deforestation, environmental and climate changes have contributed with new challenges to disease prevention and control: vectors distributions are reaching higher altitudes and latitudes increasing population at risk and changing the paradigm of poverty related disease [3-5].

The Neotropical *Leishmania* species responsible for a diversity of clinical symptoms are grouped in the two subgenera: *Viannia* and *Leishmania.* Within the *Viannia* subgenus, the species *Leishmania (Viannia) braziliensis*, *Leishmania (Viannia) panamensis* and *Leishmania (Viannia) guyanensis* are the most important causal agents of the New World tegumentary Leishmaniasis. From the *Leishmania* subgenus, the species *Leishmania (Leishmania) infantum (chagasi)* (related to Visceral Leishmaniasis) and the species of *Leishmania (Leishmania) amazonesis* and *Leishmania (Leishmania) mexicana* are responsible for the mucocutaneous, cutaneous and diffuse manifestations [6,7]. These parasite species have a wide geographic distribution from Mexico to the north of Argentina [6], with a significant increase in the last years in the number of cases in countries such as Bolivia, Brazil, Colombia and Peru [6,8-10]. The epidemiological complexity can be accompanied by a clinical difficulty when some infections, such as the one caused by *L. braziliensis,* appear in a cutaneous form and after self-healing reappear years later in a mucocutaneous form. This kind of situations makes species identification a critical step in clinical diagnosis and management.

As in most parasitic diseases, the challenge remains in having the ability to perform a rapid diagnosis of the disease and the characterization and genotyping of the different isolates obtained from infected individuals [11]. So far, the techniques typically employed to characterize and discriminate *Leishmania* species are Multi Locus Enzyme Electrophoresis (MLEE) [12] and multiple methods based on Polymerase Chain Reaction (PCR) such as Multilocus Sequence Typing (MLST) [13], PCR-Restriction Fragment Length Polymorphism (PCR-RFLP) [14], Multiplex PCR [15] and PCR followed by sequencing [16]. These methods usually use microsatellites genes as amplification targets such as: kinetoplastid DNA (kDNA), telomeric sequences, internal transcribed spacer (ITS1), Heat Shock Proteins such as HSP70 and HSP60, and genes involved in metabolic processes like Mannose Phosphate Isomerase (MPI) and 6-phosphogluconate dehydrogenase (6GPD) [11-13,17].

From this great variety of molecular targets, kDNA, ITS1, and HSP70 have been the widest employed markers due to their abundance in the number of copies across *Leishmania* genome which increases the sensitivity of the assays [18-20]. However, traditional methods have multiple disadvantages including long procedures that entail an increased risk of DNA contamination, interpretation and processing of complex data, low sensitivity, high cost and cross-reactivity [21]. To overcome these disadvantages, Real-Time PCR methods, particularly those that use SYBR Green, TaqMan probes or FRET [11-13,17,22], have become available in the last few years to ensure better reproducibility, specificity, sensitivity, and velocity during diagnosing and genotyping [23,24]. Some of these methods can distinguish between the two subgenera *L. (Leishmania*) and *L (Viannia)* at a complex level, but with many cross-reactions of human DNA and other mammals [11]. Albeit, other methods can only distinguish among the species of the *L. (Viannia)* complex [13-17].

From this perspective, it is mandatory to develop and implement a technique that allows specific, rapid, robust, and cost-effective identification of the *Leishmania* species circulating in the New World to be employed in both clinical and eco-epidemiological studies. High Resolution Melting (HRM) is a technique that has been widely used in the genotyping of bacteria, fungi, protozoan parasites and vertebrates [25-29] and could be used for identification and genotyping of these *Leishmania* species [30]. The objective of this study was to evaluate the potential of the HSP70 and ITS1 genes in genotyping six *Leishmania* species present in the New World through the use of High Resolution Melting (HRM).

Promastigotes of the reference strains *L. panamensis* (MHOM/PA/71/LS94), *L. guyanensis* (MHOM/BR/75/M4147), *L. braziliensis* (MHOM/BR/75/M2903), *L. amazonensis* (IFLA/BR/67/PH8), *L. mexicana* (MHOM/BZ/82/BEL21) and *L. infantum (chagasi)* (MHOM/TN/80/IPT1) were kindly provided by the Centro Internacional de Entrenamiento e Investigaciones Medicas (CIDEIM). Parasites were maintained in a biphasic medium of Liver Infusion (Tobie medium infused with human blood) and Triptone (LIT) supplemented with Bovine fetal serum inactivated at 20% and incubated at 25°C for 5 days to reach the late logarithmic growth phase. Aliquots of 200 μL of the LIT biphasic culture medium were obtained and then used for DNA extraction using the Roche High Pure PCR Template Preparation Kit™ following the respective instructions of the insert. The obtained DNA was quantified in a NanoDrop 2000 at a wavelength of 260 nm.

HSP70 and ITS1 genes were used given their good performance as reported by other authors [24-26]. For the HSP70 gene, primer design was carried out using the sequences available on GenBank, TriTrypDB and www.itg.be/leishmaniahsp70 of New World *Leishmania spp.* (*L. mexicana* (XM_003877072.1); *L. amazonensis* (L14605.1); *L. braziliensis* (AF291716.1); *L.guyanensis* (EU599093.1); *L. infantum (chagasi*) (XM_003392632.1); *L. panamensis* (FN395055.1)). Multiple alignments (1380 bp) were performed using the software Mega 5 [31]. Primers were designed using the software PRIMER BLAST (http://www.ncbi.nlm.nih.gov/tools/primer-blast/) from the consensus sequence obtained by the multiple alignments, with an expected amplicon size of 337 bp for all the sequences. For the amplification of ITS1 gene, the primers reported by El Tai *et. al* (2000) were used and adapted to the conditions of Real-Time PCR of this study. The expected amplicon size in this case was 300–350 pb.

Real-Time PCR was coupled together with HRM analysis using the HSP70 and ITS1 genes as targets in a Real-Time PCR system 7500 (Applied Biosystems, Inc., CA, USA) with 21 μL amplification reactions. The reaction mix contained 1X of Master Mix MeltDoctor HRM (Applied Biosystems, Inc., CA, USA), a 5 μM solution of each primers HSP70F (5’ AGG TGA AGG CGA CGA ACG 3’) and HSP70R (5’ CGC TTG TCC ATC TTT GCG TC 3’) for the amplification of HSP70 and for ITS1, LITSR (5’ CTG GAT CAT TTT CCG ATG 3’) and L5.8S (5’ TGA TAC CAC TTA TCG CAC TT 3’) [32], 6.6 μL of water and 10 ng/μL of DNA template. Real-Time PCR cycle conditions were adjusted to the following protocol: 95°C for 10 minutes (1 cycle) followed by a 40-cycles amplification (95°C for 15 seconds (denaturation) and then 60°C for 1 minute). After the Real-Time PCR, a dissociation of the amplicon was performed and followed immediately by a fusion step. The thermal profile consisted in denaturation at 95°C for 10 seconds, annealing at 60°C for 1 minute, High Resolution Melting at 95°C for 30 seconds, and a final annealing stage at 60°C for 15 seconds. During this process, the amplicons obtained from PCR were denatured prior to the development of melting curves in the inflexion point where changes in fluorescence with respect to changes in temperature (dF/dT) were recorded with a ramp of 0.1°C/seg [25]. Each DNA sample was analyzed in duplicate. Finally, the profiles of the normalized and derived fusion curves were obtained. Analyses of the obtained melting values were conducted with the software Graphpad Prism 6 (GraphPah Software, San Diego California USA, www.graphpad.com) to determine mean and standard deviations. To verify the reproducibility of the genotyping assay, this process was performed on 15 different days with the same DNA aliquots under the same conditions. The contamination controls of the study always included two controls of reaction mix without DNA (controls without templates - NTC). To evaluate the cross-reactivity, uninfected human and mouse DNA were tested and compared to the known positive range of melting temperatures for New World *Leishmania* species. Serial dilutions (factor 10) from 10^6^ to 1 parasite per mL were performed to establish the detection limit, which was defined as the minimum number of parasites necessary to generate a melting curve inside the reference values.

To determine the accuracy and reliability of the method herein reported, we analyzed 35 *Leishmania* strains obtained from humans, reservoirs and insect vectors from Colombia that were previously characterized by PCR-RFLP, monoclonal antibodies and MLEE. The HRM was applied in blind to this set of well-characterized strains and examined by duplicate. Based on this characterization, we determined the concordance between PCR-RFLP, MLEE and the HRM algorithm by means of the Kappa Index.

**Figure 1:**
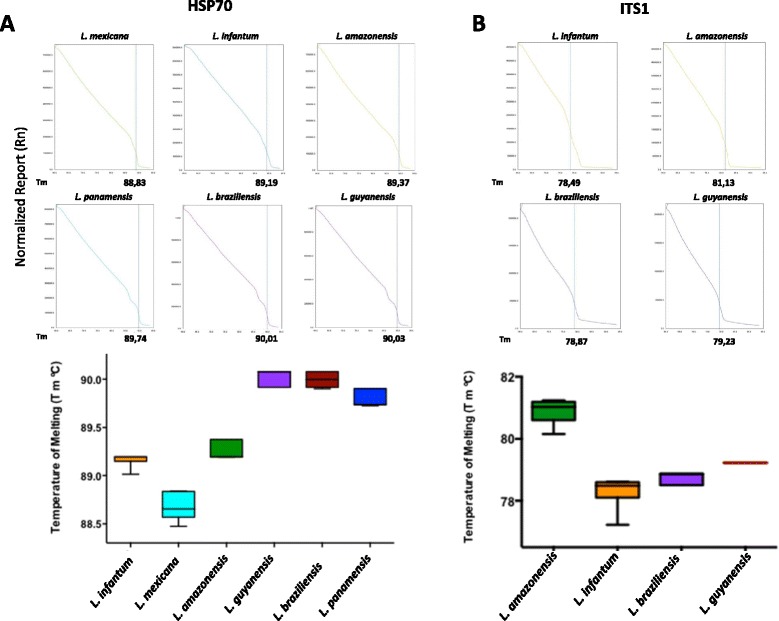
**Melting Temperature profiles and scatter plots of the HRM genotyping assay. A**. Representative normalized melting curves of each species of *Leishmania* for the genes HSP70 and ITS1.** B**. Box diagram showing the range of obtained temperatures of melting (Tm) for the curves of each species of *Leishmania.*

**Figure 2:**
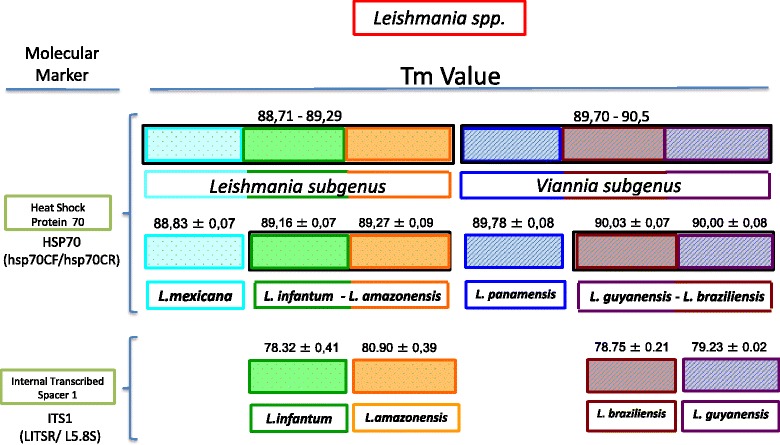
**Algorithm that shows the use of HRM assays for genes HSP70 and ITS1 in differentiating species of New World** ***Leishmania*** **.**

Primers for the HSP70 gene designed to obtain specific melting temperatures were able to discriminate the subgenera *L. (Leishmania)* and *L. (Viannia*) and the complexes *L. mexicana* from *L. infantum (chagasi) - L. amazonensis* and *L. panamensis* from *L. braziliensis - L. guyanensis* (Figure 1A). For the ITS1 gene, the obtained ranges allowed clear discrimination of the species *L. infantum (chagasi)* from *L. amazonensis* and *L. braziliensis* from *L. guyanensis* (Figure 1B). These results were used to construct the herein showed algorithm (Figure 2) which, following the amplification of HSP70 and then ITS1, can be used to discriminate six *Leishmania* species. No cross-reactivity was observed during the assays for HSP70 or ITS1 genes with uninfected human and mouse DNA. The detection limit for the HRM assay was determined on average in all examined species to be 10 parasites/mL for both markers. Based on the designed algorithm, we tested 35 well-characterized *Leishmania* strains. We were able to obtain reliable Tm curves for each strain that were in accordance with the ranges found in the reference strains (Table 1). We calculated the Kappa Index considering PCR-RFLP/MLEE as the gold standard and the results showed a High concordance between HRM and PCR-RFLP/MLEE (KI = 1.0).

**Table 1:** **Comparison of HRM assay and PCR-RFLP/MLEE in the set of well-characterized** ***Leishmania*** **strains**

**Sample name**	**Geographical origin**	**Host**	**PCR-RFLP/MLEE**	**HSP70**	**Tm**	**ITS1**	**Tm**
6	Meta/Colombia	*Homo sapiens*	*L. guyanensis*	*L. guyanensis/L. braziliensis*	90,08	*L. guyanensis*	79,22
7	Santander/Colombia	*Lutzomyia umbratilis*	*L. guyanensis*	*L. guyanensis/L. braziliensis*	90,07	*L. guyanensis*	79,21
71	Boyaca/Colombia	*Lutzomyia umbratilis*	*L. guyanensis*	*L. guyanensis/L. braziliensis*	90,07	*L. guyanensis*	79,23
875	Santander/Colombia	*Homo sapiens*	*L. infantum (chagasi)*	*L. guyanensis/L. braziliensis*	90,07	*L. chagasi*	79,21
924	Santander/Colombia	*Homo sapiens*	*L. panamensis*	*L. panamensis*	89,91	*L. panamensis*	-
113	Meta/Colombia	*Homo sapiens*	*L. braziliensis*	*L. guyanensis/L. braziliensis*	90,08	*L. braziliensis*	78,77
12	Boyaca/Colombia	*Homo sapiens*	*L. braziliensis*	*L. guyanensis/L. braziliensis*	90,09	*L. braziliensis*	78,55
13	Boyaca/Colombia	*Homo sapiens*	*L. guyanensis*	*L. guyanensis/L. braziliensis*	90,06	*L. guyanensis*	79,22
15	Boyaca/Colombia	*Didelphis marsupialis*	*L. panamensis*	*L. panamensis*	89,73	*L. panamensis*	-
19	Meta/Colombia	*Lutzomyia spinicrassa*	*L. braziliensis*	*L. guyanensis/L. braziliensis*	90,09	*L. braziliensis*	78,77
20	Meta/Colombia	*Lutzomyia trapidoi*	*L. panamensis*	*L. panamensis*	89,73	*L. panamensis*	-
21	Meta/Colombia	*Lutzomyia trapidoi*	*L. panamensis*	*L. panamensis*	89,83	*L. panamensis*	-
23	Meta/Colombia	*Lutzomyia trapidoi*	*L. panamensis*	*L. panamensis*	89,73	*L. panamensis*	-
86	Santander/Colombia	*Homo sapiens*	*L. infantum (chagasi)*	*L. infantum/L. amazonensis*	89,14	*L. chagasi*	78,33
87	Santander/Colombia	*Homo sapiens*	*L. infantum (chagasi)*	*L. infantum/L. amazonensis*	89,17	*L. chagasi*	78,56
94	Santander/Colombia	*Homo sapiens*	*L. infantum (chagasi)*	*L. infantum/L. amazonensis*	89,19	*L. chagasi*	78,55
094b	Antioquia/Colombia	*Didelphis marsupialis*	*L. mexicana*	*L. mexicana*	88,75	*L. mexicana*	-
245	Antioquia/Colombia	*Homo sapiens*	*L. amazonensis*	*L. infantum/L. amazonensis*	89,19	*L. amazonensis*	80,61
280	Antioquia/Colombia	*Homo sapiens*	*L. amazonensis*	*L. infantum/L. amazonensis*	89,19	*L. amazonensis*	80,77
916	Antioquia/Colombia	*Lutzomyia trapidoi*	*L. panamensis*	*L. panamensis*	89,79	*L. panamensis*	-
916	Antioquia/Colombia	*Lutzomyia trapidoi*	*L. panamensis*	*L. panamensis*	89,73	*L. panamensis*	-
984	Antioquia/Colombia	*Homo sapiens*	*L. braziliensis*	*L. guyanensis/L. braziliensis*	90,04	*L. braziliensis*	78,77
985	Antioquia/Colombia	*Lutzomyia spinicrassa*	*L. braziliensis*	*L. guyanensis/L. braziliensis*	90,08	*L. braziliensis*	78,89
986	Meta/Colombia	*Homo sapiens*	*L. braziliensis*	*L. guyanensis/L. braziliensis*	90,09	*L. braziliensis*	78,91
990	Meta/Colombia	*Homo sapiens*	*L. guyanensis*	*L. guyanensis/L. braziliensis*	90,04	*L. guyanensis*	79,22
094b	Meta/Colombia	*Homo sapiens*	*L. infantum (chagasi)*	*L. infantum/L. amazonensis*	89,22	*L. chagasi*	78,67
094b	Meta/Colombia	*Homo sapiens*	*L. infantum (chagasi)*	*L. infantum/L. amazonensis*	89,29	*L. chagasi*	78,66
101	Boyaca/Colombia	*Homo sapiens*	*L. amazonensis*	*L. infantum/L. amazonensis*	89,14	*L. amazonensis*	81,11
123	Boyaca/Colombia	*Homo sapiens*	*L. braziliensis*	*L. guyanensis/L. braziliensis*	90,02	*L. braziliensis*	78,66
157	Santander/Colombia	*Homo sapiens*	*L. guyanensis*	*L. guyanensis/L. braziliensis*	90,04	*L. guyanensis*	79,22
146	Santander/Colombia	*Homo sapiens*	*L. guyanensis*	*L. guyanensis/L. braziliensis*	90,01	*L. guyanensis*	79,22
147	Santander/Colombia	*Didelphis marsupialis*	*L. mexicana*	*L. mexicana*	88,37	*L. mexicana*	-
222	Santander/Colombia	*Homo sapiens*	*L. panamensis*	*L. panamensis*	89,82	*L. panamensis*	-
2698	Santander/Colombia	*Homo sapiens*	*L. panamensis*	*L. panamensis*	89,81	*L. panamensis*	-
1453	Santander/Colombia	*Lutzomyia trapidoi*	*L. panamensis*	*L. panamensis*	89,88	*L. panamensis*	-

Currently, the methods employed for the discrimination of New World *Leishmania* species are laborious, subjective and often lacking of specificity [21]. Therefore, it is crucial to count on a low-cost, effective and rapid tool that allows an accurate detection and identification of the *Leishmania* species present in the New World or at least the most prevalent. Herein, the proposed algorithm allowed identifying *L. mexicana, L. infantum (chagasi), L. amazonensis, L. panamensis, L. braziliensis* and *L. guyanensis* with a Real-Time PCR coupled with HRM analysis after the amplification of HSP70 gene and the subsequent amplification of ITS1 gene. The HRM methodology is considered as an excellent option to reduce some problems of the previously molecular techniques used for *Leishmania* genotyping such as the need of modified oligonucleotides, low accuracy, limited high-throughput applications, etc. [33] since this is a closed-tube assay (does not require additional precautions to prevent crossover of PCR products), avoids sequencing processes, laborious procedures and high costs as well as offering robust and high efficiency in its results [25,34]. On average, this type of test has been calculated to be three times faster and five times cheaper than other types of analysis such as MLST and RFLPs [17].

Additionally, our algorithm helps to differentiate between *L. (L). mexicana* and *L. (L). amazonensis*, two species that in previous studies could not be identified using HRM tests [11,17]. This differentiation is important, and although *L. (L.) amazonensis* infections usually exhibit a milder form of the disease, it can also cause diffuse forms that do not respond adequately to existing treatments [35]. On the other hand, our algorithm can also differentiate between *L. braziliensis* (the main etiologic cause of cutaneous Leishmaniasis) and *L. guyanensis* (with high prevalence in the Amazon region). This is of great importance due to its eco-epidemiological implications and the close genetic relationship of the members of the subgenus *Viannia* that has represented a limited number of techniques that can be used to correctly identify them [36]. As well, HSP70 and ITS1 showed a higher resolution in characterizing the members of the subgenera *Viannia* and *Leishmania* than other genes. For example, markers like kDNA can only discriminate between fewer members of these subgenera but are nonetheless used in many assays as a gold standard for diagnosis of Old and New World Leishmaniasis due to its high sensitivity [11,17].

The results of our study support the idea that genes such HSP70 and ITS1 are good candidates for genotyping neotropical *Leishmania* species, and that these genes allow detection of genetic variability in molecules associated with immunological processes (HSP70) and confer a high probability of being detected due to their high number of copies [35]. Similarly, regarding specificity, this method does not cause mammalian DNA cross-reactions like has been previously noted in studies using HRM [11]. This fact makes HRM a good candidate for identifying *Leishmania* in epidemiological studies. Also, the technique is reproducible, and this was retained even though the tests were duplicated and performed on different days, albeit under the same conditions of DNA and reactive concentrations.

We decided to apply this algorithm to a set of well-characterized strains by the gold standard method (PCR-RFLP/MLEE). The findings allowed observing that HRM was accurate enough to discriminate six Neotropical species and was 100% concordant with PCR-RFLP/MLEE which suggests its suitability in the application to a broad number of epidemiological questions. Another important feature of our assay besides its reproducibility was a tailored sensitivity obtained where the method is able to detect at least 10 parasites/mL and is considered a remarkable value of sensitivity. These advantages demonstrate that this method has a tremendous potential for the identification of New World *Leishmania* species and is a suitable choice for identification and characterization. Nonetheless, it is important to point out that the *Leishmania* strains employed in this implementation were isolated from Colombian hosts and unbiased range of *Leishmania* diversity was not considered. Therefore, we suggest that this novel method is implemented in strains from Meso-America in order to validate the feasibility of the technique.

## Conclusions

In conclusion, the suggested HRM assay in this study is a reliable, robust and reproducible technique that allows the correct genotyping of six New World *Leishmania* species, in a rapid way (it does not require post-PCR treatments), little labor and low cost (around 7.5 USD) with a potential applicability in different areas of molecular epidemiology of Leishmaniasis such as host-parasite relationships, eco-epidemiology and infection dynamics in urban and wild areas. We encourage the scientific community from *Leishmania* endemic areas to test this novel approach in order to fully validate its reliability at a continental level.
